# Assessing the Incidence of Snakebites in Rural Gabon—A Community-Based, Cross-Sectional Pilot Survey

**DOI:** 10.3390/tropicalmed9040068

**Published:** 2024-03-23

**Authors:** Saskia Dede Davi, Anita Lumeka, Teite Rebecca Hildebrandt, Lilian Rene Endamne, Cedric Otchague, Dearie Glory Okwu, Rica Artus, Friederike Hunstig, Rella Zoleko Manego, Jörg Blessmann, Peter G. Kremsner, Bertrand Lell, Ghyslain Mombo-Ngoma, Selidji Todagbe Agnandji, Michael Ramharter, Benno Kreuels

**Affiliations:** 1 Centre of Tropical Medicine, Bernhard-Nocht Institute for Tropical Medicine & I. Department of Medicine, University Medical Center Hamburg-Eppendorf, 20359 Hamburg, Germany; saskia.davi@bnitm.de (S.D.D.); teite.hildebrandt@uni-rostock.de (T.R.H.);; 2 Centre de Recherches Médicales de Lambaréné, Lambarene BP 242, Gabon; anlumeka@yahoo.com (A.L.); otchaguecedric@gmail.com (C.O.); ghyslain.mombo-ngoma@bnitm.de (G.M.-N.); agnandjis@cermel.org (S.T.A.); 3 German Centre for Infection Research (DZIF), Partner Site Hamburg-Luebeck-Borstel-Riems, 20359 Hamburg, Germany; 4 Research Group Snakebite Envenoming, Department of Implementation Research, Bernhard-Nocht Institute for Tropical Medicine, 20359 Hamburg, Germanyblessmann@bnitm.de (J.B.); 5 Institut für Tropenmedizin, Universitätsklinikum Tübingen, 72016 Tübingen, Germany; 6 Department of Medicine I, Division of Infectious Diseases and Tropical Medicine, Medical University of Vienna, 1090 Vienna, Austria; 7 Research Group Drug Implementation, Department of Implementation Research, Bernhard-Nocht Institute for Tropical Medicine, 20359 Hamburg, Germany; 8 Division for Tropical Diseases, I. Department of Medicine, University Medical Center Hamburg-Eppendorf, 20359 Hamburg, Germany

**Keywords:** snakebites, snakebite envenomation, Gabon, sub-Saharan Africa, neglected tropical diseases

## Abstract

Snakebite envenoming (SBE) is a neglected tropical disease (NTD). Community-based studies from sub-Saharan Africa are urgently required as data on the incidence are scarce. This study aimed to determine the lifetime prevalence of snakebites in rural Gabon by preparing the conduct of a larger regional survey. A cross-sectional community-based epidemiological survey in Sindara, Ngounie province, was conducted. Households were interviewed about the history of snakebites of household members to calculate lifetime prevalence. In addition, the average annual incidence rate per 100,000 over the last 5 years was calculated. A total of 771 inhabitants were enrolled, of which 5 (0.65%; 95% confidence interval (95% CI: 0.2–1.5%)) were victims of snakebites. Over the past 5 years, annual incidence was 77 bites per 100,000 (95% CI: 0–620). This study provides a first rough estimate of the incidence of SBE from rural central Gabon, demonstrating the importance of this NTD. **Key Contribution:** The estimated annual incidence of snakebites found was 77 per 100,000. Snakebites occurred mainly during agricultural activities.

## 1. Introduction

The World Health Organisation (WHO) estimates that 81,000–138,000 deaths are caused by snakebite envenoming (SBE) annually and that about three times as many amputations and other permanent disabilities are attributable to snakebite envenoming [1]. The burden of this disease is the highest in Africa, Asia and Latin America. In the 1990s, Chippaux et al. estimated an annual 1,000,000 snakebites in Africa, leading to an estimated 500,000 envenomation, 200,000 hospitalisations and 20,000 deaths per year [2]. More recently, Kasturiratne et al. [3] calculated an incidence of snakebite envenoming of 20–53/100,000 persons with a mortality rate of 0.3–3.4/100,000 for central sub-Saharan Africa and 20,000–94,000 deaths annually worldwide. The wide range of estimates reflects the high level of uncertainty in these numbers. The regions where snakebites occur are primarily resource-limited countries with weak health and surveillance systems. In these countries, agricultural workers and children from poor communities in rural settings are the most exposed to snake–human interaction [4].

Gabon has 30 venomous snake species, of which most are only mildly venomous [5]. Anecdotally, three snakes are most likely responsible for cases of severe envenoming, the Gaboon Viper (*Bitis gabonica*), the Forest Cobra (*Naja melanoleuca*) and Jameson’s Mamba (*Dendroaspis jamesoni*).

However, the incidence of and mortality from venomous snakebites are poorly understood.

Snakebites are not routinely reported within the health system, and data collected by the infectious disease program on SBE is incomplete; however, a nationwide DHIS2 system is currently being implemented and will include this NTD. In a retrospective study, the authors reported 157 admissions for snakebite to the Intensive Care Unit of a Libreville hospital between 1998 and 2001 [6]. Of these, 27 individuals showed signs of envenoming. This study was performed in a referral hospital in the capital and is not representative of the situation in Gabon. The true burden of snakebite envenoming is almost certainly underestimated as most snakebites occur in rural areas, and treatment-seeking behaviour of affected individuals, influenced by socioeconomic and cultural factors, leads to a preference for traditional practices so that victims often do not reach the formal healthcare system [7,8,9]. Community-based studies on the incidence of snakebites are urgently needed to close the gap in our understanding of the incidence of SBE and provide more accurate estimates to inform policy and guide resource allocation [10]. To date, epidemiological data on snakebite envenoming in rural Gabon are unavailable. This cross-sectional community-based epidemiological survey was conducted to provide the first data on the incidence of snakebites in rural Gabon and to prepare for a larger regional survey in central Gabon.

## 2. Results

### 2.1. Study Population

**Figure 1:**
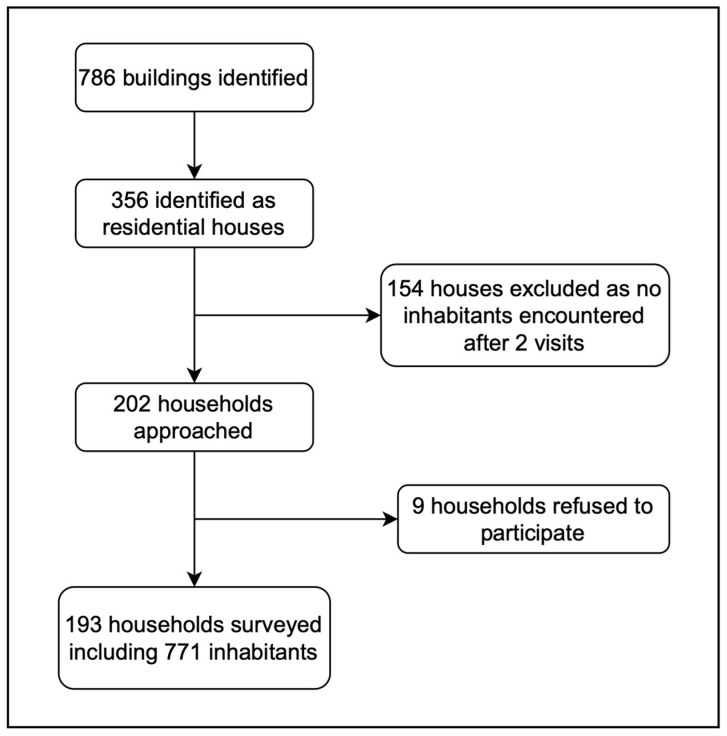
Flowchart demonstrating recruitment to the study.

In total, 786 buildings were identified in Sindara, of which 356 (45.3%) were categorised as potentially inhabited residential buildings. Of these buildings, 154 (43.3%) were approached at least twice but had to be excluded due to the absence of inhabitants. In total, the inhabitants of 202 (56.7%) households were approached for participation in the survey. Nine households (4.4%) refused to participate in the study. In the 193 enrolled households, we counted 771 inhabitants, of which 386 (50.1%) were female (Figure 1). The median household size was three individuals, ranging from 1 to 19 (IQR 2–6). The median age of the study participants was 34 years (IQR: 10–52). A total of 47% (*n* = 362) were younger than 18 years, and 15.4% (*n* = 119) were under 5 years of age.

### 2.2. Prevalence of Snakebites and Risk Factors

Five of the 771 participants (0.65%; 95% confidence interval (95% CI): 0.2–1.5%) indicated that a snake had bitten them in their lifetime. Among the five victims, one had been bitten within the previous year, two had been bitten between one and five years ago, and two had been bitten more than five years ago. Based on these data, we calculated an average annual incidence rate of 77 per 100,000 (95% CI 0–620) over the past five years.

A green snake bit two participants, and three were bitten by a black/brown snake. The bites occurred mainly during agricultural activities such as work on the plantation, hunting and weeding (*n* = 4), while one bite occurred on a construction site. Reported symptoms were bleeding (*n* = 4) and swelling (*n* = 1). None reported neurotoxic signs of envenoming.

Two victims went to a higher care hospital in Lambaréné and Mouila, while three exclusively sought the help of a traditional healer. In this survey, there were no reports of deaths caused by snake bites.

Several respondents indicated that domestic animals, especially chickens, were frequently attacked by snakes. However, this information was not systematically collected in the study.

## 3. Discussion

Understanding the epidemiology of snakebites and envenomation, as well as the clinical consequences that come with it, is crucial to developing treatment guidelines, planning prevention programs and allocating resources. However, data, especially from sub-Saharan Africa, are scarce [11]. In this study, we show that 0.65% (95% CI: 0.2–1.5%) of inhabitants of a rural community in Gabon had been bitten by a snake in their lifetime. The annual incidence was estimated at around 77 bites per 100,000. While the data collected in this study stem from a limited sample of 771 individuals, leading to relatively large confidence intervals around our point estimates, these are comparable to those from larger studies from Cameroon [12] or Ghana [8]. Estimates are also similar to the reported overall incidence in sub-Saharan Africa of 20–53/100,000 persons in a systematic review by Kasturiratne et al. [3]. Importantly, it was shown that snakebites in the study region occurred mainly during agricultural activities, which is similar to other studies [13], indicating a possible target for prevention measures [10].

Our results show that most victims seek care from traditional healers and not the formal governmental healthcare system. While this health-seeking behaviour is largely influenced by local factors, this fits well with previous reports [7,9,14]. Consequently, healthcare facility-based data on SBE strongly underestimate the true incidence and the sequelae, disabilities and deaths relied on SBE in an area [13]. In addition, preferentially severe cases tend to seek health care in the hospitals operating within the formal healthcare sector. Thus, well-designed community-based studies of sufficient sample size are urgently needed to accurately determine the true burden of disease [10]. In addition, further studies to examine the knowledge, attitudes and practices of traditional healers and their collaboration with other medical facilities are needed to understand healthcare-seeking behaviour and improve referral pathways [15].

There are several limitations to our study. First, the sample size of this pilot survey is limited; therefore, the confidence intervals of the estimated incidence are large. The aim of this survey was, however, not only to provide a first estimate of incidence but also to gather initial data to inform a sample size calculation for a subsequent larger survey on a regional level in central Gabon. Second, the geographical region that was covered in this survey was limited. Therefore, the findings may not be generalisable to the other areas of Gabon. Distances to towns that provide health care might influence health seeking behaviour, while the location of the settlement (along the road, along the river, near lakes) and common daily activities (farming, fishing, hunting) may influence the risk of bites. In the subsequent study, we plan to consider regional differences within the district. Here, we identified different locations that we classified as urban (the town of Lambaréné), rural (along main roads) and very remote areas without access to roads. The population in these areas may have different risks for snakebites and will be sampled proportionally, providing reliable estimates representative of a larger region of Gabon.

Several lessons were found from the sampling and application of the questionnaire. First, it should be noted that a surprisingly high number of households were found to be absent (43%), even though fieldworkers collected data from 9:00 to 18:00 on weekdays. How this may have influenced the estimates obtained in this study is unclear. Apart from the resulting lower sample size, a bias may have been introduced in both ways. Snakebite victims may have been more likely to stay at home due to resulting disabilities, which could lead to an overestimation. However, no permanent disabilities were recorded in the patients with bites. On the other hand, persons working in the forest may be more likely to be missed, leading to an underestimation of bites. It is also possible that some houses were only used on weekends or as seasonal houses by inhabitants of Lambarene. We, therefore, see the need to adapt the visit schedule to the sampled households to increase the probability of finding people present at their homes. This includes weekends, early morning or late evening visits to include all eligible participants.

Second, many respondents indicated that domestic animals, especially chickens, were frequently attacked by snakes, even if these data were not collected systematically in this pilot study. In our opinion, a one-health approach to assessing snakebite envenoming would be helpful, among others, to estimate the economic impact on affected families. We, therefore, decided to include questions on the ownership of animals and the frequency of attacks on animals in our subsequent study.

Third, it was noted that some individuals reported having been bitten by a snake while, on further questioning, were unable to provide additional details on the bite. In these cases, when witnesses could not confirm bites, we have to assume that the bites may not have occurred. To minimise the risk of including such bites, plausibility checks referring to the situation and patient-related circumstances must be introduced. These could include that for every bite, the snake should have been seen by the victim or another person (during daytime) and that the situation of the bite and the subsequent actions can be clearly described.

Finally, very little is known about the snakes responsible for bites and the resulting clinical syndromes in Gabon. We therefore included more detailed questions on the responsible snake in our subsequent study and aim to analyse cases presenting to health facilities to assess the clinical syndromes and treatment practices, as well as the availability and use of antivenom in the region.

In summary, our study provides the first rough estimate of the incidence of SBE in rural central Gabon and demonstrates that this NTD is important in the country. A larger survey based on an adapted methodology, including surveys in the healthcare system and among traditional healers, is currently being conducted. It will provide urgently needed, more accurate data on the incidence of SBE in Gabon, including healthcare-seeking behaviour, clinical consequences and potential targets for preventive measures. These data will help to guide policy, the distribution of resources, and training measures, as well as community engagement measures. Ultimately, this will help to improve the treatment of patients with snakebite envenoming and reduce the incidence of snakebites in the country.

## 4. Materials and Methods

### 4.1. Study Setting

**Figure 2:**
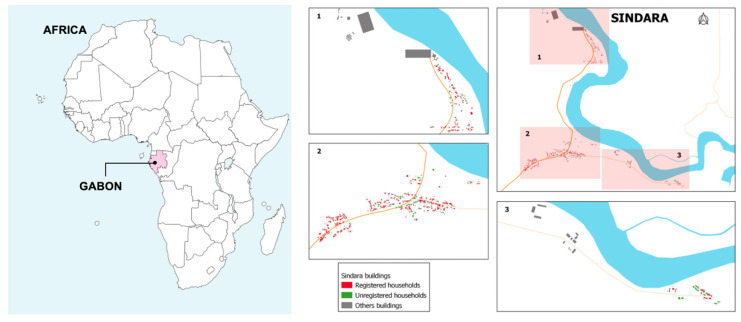
Map of the study area showing the village of Sindara. The map of Africa was created with mapchart.net.

The study was conducted from January 2022 to April 2022 in the village Sindara (Figure 2), located in the Department of Tsamba-Magotsi, in the province of Ngounié in central Gabon. The village is 90 km southeast of Lambarene and approximately 28 km from Fougamou. Sindara is approximately 230 m above sea level and is fully surrounded by tropical rainforest. The Ngounié River, the last and second most important tributary of the Ogooue River, flows through Sindara. The climate is characterised by equatorial humidity, greater than 80%, with an average daily temperature of 30 °C. There are two rainy and two dry seasons [16]. Malaria is a holoendemic and parasitic disease, and human filariasis, soil-transmitted helminths, and schistosomiasis are also highly prevalent [17]. Houses are typically constructed from wood, occasionally clay, with metal roofs. Floors are commonly clay, and dwellings often lack tight seals, allowing easy entry for snakes and reptiles. Public lighting is absent, and outhouses serve as toilets. The closest health facilities providing advanced medical care are in Mouila, approximately 130 km, and in Lambarene, around 90 km from Sindara. The population’s main commercial activities are hunting, fishing, and subsistence farming, with fields located mainly within the rainforest. A demographic and health surveillance system (DHSS) was recently established in the community of Sindara by the Institut de Santé de Sindara (ISSA), which is a satellite site of the Centre de Recherches Médicales de Lambaréné (CERMEL).

### 4.2. Study Design and Procedures

#### Demographic and Health Surveillance System

Data for this study were collected within the framework of a baseline assessment of a newly set-up DHSS. The study team then visited all potentially inhabited residential buildings on weekdays from 9:00 to 18:00. If a building was found empty but still looked inhabited, it was visited for a second time. If no one was found to be present after two visits, the house was excluded from the DHSS and the survey.

All households were invited to participate in this survey and were visited by a field worker. Everyone willing to participate was eligible. Collected data about baseline sociodemographic information, i.e., the number of individuals per household, date of birth and sex, were entered in an electronic form on a handheld tablet.

### 4.3. Questionnaire Design

We adapted a questionnaire previously administered to study participants in Laos and Vietnam to assess the incidence and consequences of snakebites in the study region [18]. This questionnaire was shown to members of the local study team to assess for cultural appropriateness and logistical practicality. The major adjustment to the questionnaire was that individuals were asked about the colour of the snake (brown/black or green) instead of the snake species, as it was deemed unlikely that it would be possible to correctly identify the snake in question.

### 4.4. Data Collection

Fieldworkers, trained twice for the study procedures, administered the resulting questionnaire on snakebites. The administration of the questionnaire for the whole DHSS, including the section related to snakebites, took about 30 min per household. All participants willing to participate were asked whether a snake had bitten them at any point in their life or if a snake had bitten anyone else in the household. If both questions were answered with “no”, no further questions on snakebites were asked.

In the case of a snakebite report, further data on the timepoint of the bite, the type of snake, the situation during which the snakebite occurred, the location of the bite, symptoms, how and where the snakebite was treated, and the clinical outcome of the snakebite were collected. In case a different household member had been bitten, an attempt was made to speak directly with the victim. If the victim was unavailable, relevant data were collected from the person interviewed. Ethical approval for this study was obtained from the Institutional Ethical Committee of Centre de Recherches Médicales de Lambaréné (CEI-008/2021). The head of each household gave written informed consent before any study procedure.

### 4.5. Data Analysis

Data on snakebites were extracted from the online database of the survey (PostgreSQL 14) in a pseudonymised form and analysed using Stata Statistical Software (Release 17, College Station, TX, USA: StataCorp). Descriptive demographics were displayed as percentages, medians (interquartile range) or means (with standard deviations). The lifetime prevalence of snakebites was calculated as the proportion of participants who reported having been bitten at any time. The average annual incidence of snakebites in the 5 years preceding the survey was calculated as the proportion of participants who indicated to have been bitten within the last five years divided by five and then extrapolated to a rate per 100,000 individuals per year.

## Data Availability

The data generated to compile this manuscript are available from the authors upon reasonable request.
